# Association Between the Phase Angle and Function Recovery in Patients With Hip Fractures

**DOI:** 10.7759/cureus.96754

**Published:** 2025-11-13

**Authors:** Hironori Unno, Takahiro Hasegawa, Shinya Takigawa, Masayoshi Sato, Masahiro Hasegawa

**Affiliations:** 1 Orthopaedics, Iga City General Hospital, Iga, JPN; 2 Orthopaedic Surgery, Mie University Graduate School of Medicine, Tsu, JPN

**Keywords:** bia, fim, hip fracture, nutrition, phase angle, sarcopenia

## Abstract

Background: Patients with hip fractures frequently present with malnutrition, and proper nutritional management is recognized as an important factor in their clinical care.

Objective: We aimed to clarify whether the preoperative phase angle could predict postoperative functional recovery and to identify the impact of undernutrition on clinical outcomes in patients with hip fractures.

Methods: For 100 hip fracture patients, serum albumin level and muscle mass on the admission day were recorded. Muscle mass was estimated using plain computed tomography (CT) images with the skeletal muscle index (SMI). All participants were subjected to bioelectrical impedance analysis (BIA), and the phase angle was calculated at admission. Functional Independence Measure (FIM) scores on admission and at two weeks postoperatively and ΔFIM (the difference between the FIM score on admission and the score at two weeks postoperatively) were determined. Correlations among the phase angle, serum albumin level, SMI, and FIM scores were assessed.

Result: Positive correlations were observed between the phase angle with both serum albumin level at admission (R=0.53, p<0.001) and SMI (R=0.43, p<0.001). Serum albumin level at admission and the phase angle were positively correlated with both the FIM score at two weeks postoperatively and ΔFIM. From multiple regression analysis with the FIM score at two weeks postoperatively or ΔFIM as the dependent variable, and the phase angle, serum albumin level at admission, and SMI as three independent variables, only the phase angle showed significant correlations with the FIM score at two weeks postoperatively (p<0.001) and ΔFIM (p<0.05).

Conclusions: The preoperative phase angle could predict postoperative functional recovery and identify the impact of undernutrition and sarcopenia on clinical outcomes in patients with hip fractures.

## Introduction

Hip fractures are a global public health problem that results in hospitalization, disability, and death [1]. As the world population ages, the number of hip fractures increases, and 6.3 million people are expected to suffer from hip fractures globally by 2050 [2]. Hip fractures in geriatric patients have a negative impact on functional status and quality of life and are associated with high mortality [3,4].

Patients with hip fractures experience multiple geriatric nutritional problems on admission, which often include undernutrition and sarcopenia. Recent studies have shown poor outcomes in patients with hip fractures who had a poor preoperative nutritional status. Low preoperative serum albumin, a common biomarker for nutrition, has been used to predict disability, significantly higher in-hospital mortality, and specific postoperative complications [5]. However, Ishida et al. showed that serum albumin level was not a good nutritional marker for patients with burns [6].

In recent years, bioelectrical impedance analysis (BIA), which can estimate body composition by measuring body impedance, has been widely used in various clinical settings. BIA is a noninvasive method that estimates body composition by measuring resistance and reactance of body tissues to a small electrical current. The phase angle can be calculated quickly and easily by low-cost bioelectrical impedance analyzers, such as the arctangent of the bioelectrical reactance and resistance [7]. Phase angle represents the difference in phase between the electrical current and voltage that arises from the properties of the cell membrane. It has been regarded as a marker of cellular condition: lower values are linked to reduced membrane integrity or cell death, whereas higher values indicate preserved cellularity, intact membranes, and superior cell performance [8]. Consequently, phase angle has been suggested as a prognostic index for various clinical outcomes such as nutritional impairment, diminished functional capacity, postoperative complications, and mortality [9].

We hypothesized that postoperative functional recovery in patients with hip fractures would be related to the phase angle. This study aimed to clarify whether the preoperative phase angle could accurately predict postoperative functional recovery and to identify the impact of undernutrition and sarcopenia on clinical outcomes in patients with hip fractures. Therefore, we investigated the associations among the phase angle, serum albumin level, skeletal muscle mass on admission, and functional recovery in patients with hip fractures.

## Materials and methods

We retrospectively reviewed patients with hip fractures in our hospital between 2020 and 2021. All consecutive patients aged >65 years old with hip fractures who were treated surgically were included in the study. We identified 100 patients (21 men, 79 women) with a mean age of 84.9 years (range: 66-99 years), mean body weight of 47.3 kg, and mean body mass index (BMI) of 20.7 kg/m^2^. The underlying injury was a femoral neck fracture in 46 patients and a trochanteric fracture in 54 patients. All patients were treated surgically. Patients with femoral neck fractures were classified according to the Garden classification. Internal fixation with cannulated cancellous screws was performed for type I or II fractures, while type III or IV fractures were generally managed with hemiarthroplasty. Trochanteric fractures were stabilized using proximal femoral nails. Postoperative rehabilitation was initiated without delay and continued throughout hospitalization. All patients were permitted to bear full weight beginning on the first postoperative day. A standardized rehabilitation protocol was applied, progressing from wheelchair transfer to assisted ambulation with parallel bars, then walker, cane, and eventually independent walking. At discharge, patients either returned home or were transferred to another facility for ongoing care, if required.

**Figure 1:**
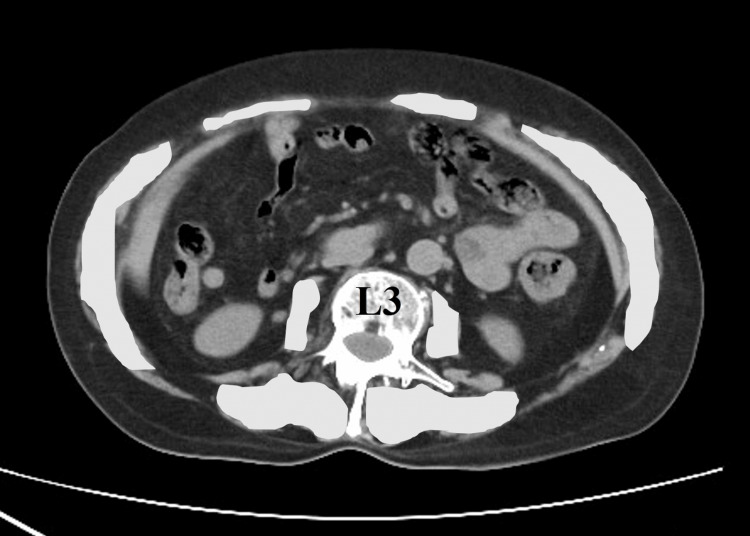
Skeletal muscle index (SMI) Skeletal muscle index (SMI) was calculated from the cumulative volume of the intra‐abdominal musculature at the level of the third lumbar vertebra (cm^2^) divided by the square of height (m^2^).

Serum albumin level and muscle mass on the admission day were recorded for each patient. Muscle mass was estimated using plain computed tomography (CT) images. Skeletal muscle index (SMI) is most often calculated from the cumulative volume of the intra-abdominal musculature on a CT image slice, generally at the level of the third lumbar vertebra, and normalized by the square of the patient’s height in meters (Figure 1).

All participants were subjected to BIA using a Direct Segmental Multi-Frequency Bioelectrical Impedance Analyzer (DSM-BIA, Inbody S10, Japan). BIA was performed on the hospital admission day of the participant. All BIA parameters were obtained using a standard montage of the outer and inner electrodes on the right hand and foot while the patients lay down with their legs apart. The phase angle was derived according to the formula: phase angle (°) = arctangent (reactance/resistance) × (180/π). The BIA system employed in this study was capable of determining phase angle values for the entire body, as well as for specific regions such as the trunk.

Patients were evaluated according to the outcomes of the rehabilitation process using the Functional Independence Measure (FIM) scores on the admission day and at two weeks postoperatively, and ΔFIM (the difference between the FIM score on admission and the score at two weeks postoperatively). FIM is a widely used scale that evaluates a patient’s level of independence in performing activities of daily living. Higher FIM scores indicate greater functional independence, whereas lower scores reflect severe functional impairment and dependence on assistance for basic daily activities.

This research has been approved by the Institutional Review Board (IRB) of the authors’ affiliated institutions.

Statistical analysis was performed with the Statistical Product and Service Solutions (SPSS; IBM SPSS Statistics for Windows, Armonk, NY) software. Statistical significance was set at p<0.05. Correlation analysis was performed using the Pearson product-moment correlation. Based on the correlation coefficients, the coefficient of determination was calculated as R^2^.

## Results

**Figure 2:**
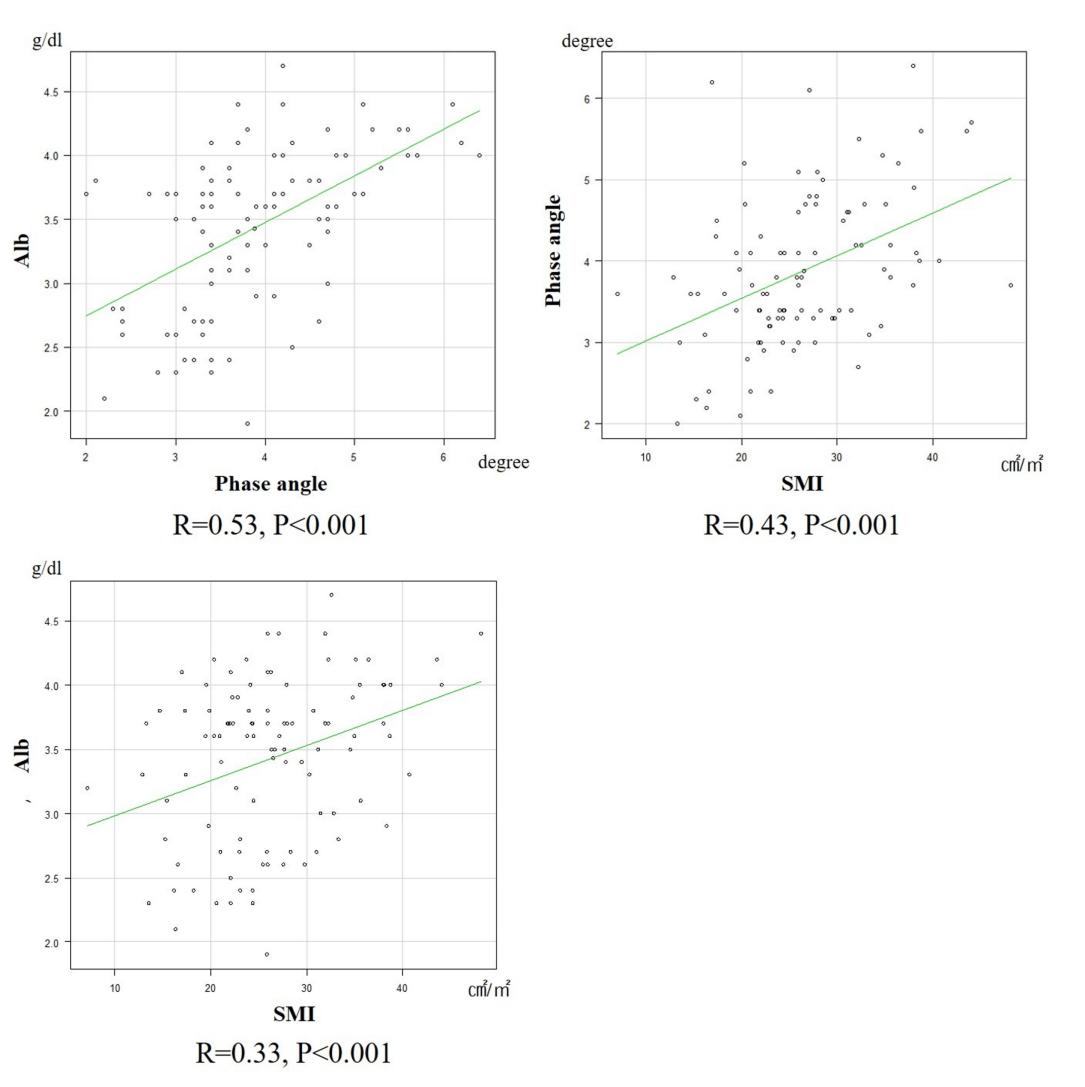
Correlations among serum albumin level, skeletal muscle index (SMI), and the phase angle

The mean serum albumin level on admission was 3.4±0.6 g/dL, the mean SMI was 26.5±7.8 cm^2^/m^2^, and the mean phase angle was 3.9±0.9° (Table 1). Positive correlations were observed between the phase angle with both serum albumin level at admission (R=0.53, p<0.001) and SMI (R=0.43, p<0.001). Moreover, a positive correlation was observed between serum albumin level at admission and SMI (R=0.33, p=0.00087) (Figure 2). 

**Table 1:** Demographic data of participants

Patients	100
Gender (male/female)	21/79
Age	84.9±8.0
Body weight (kg)	47.3±10.5
Body mass index (kg/m^2^)	20.7±3.44
Fracture type (femoral neck /trochanteric)	46/54
Serum albumin level (g/dL)	3.4±0.6
Skeletal muscle index (cm^2^/m_2_)	26.5±7.8
Phase angle (°)	3.9±0.9
FIM at admission	24.3±13.4
FIM at 2 weeks	42.0±24.1
ΔFIM	17.8±17.1

**Figure 3:**
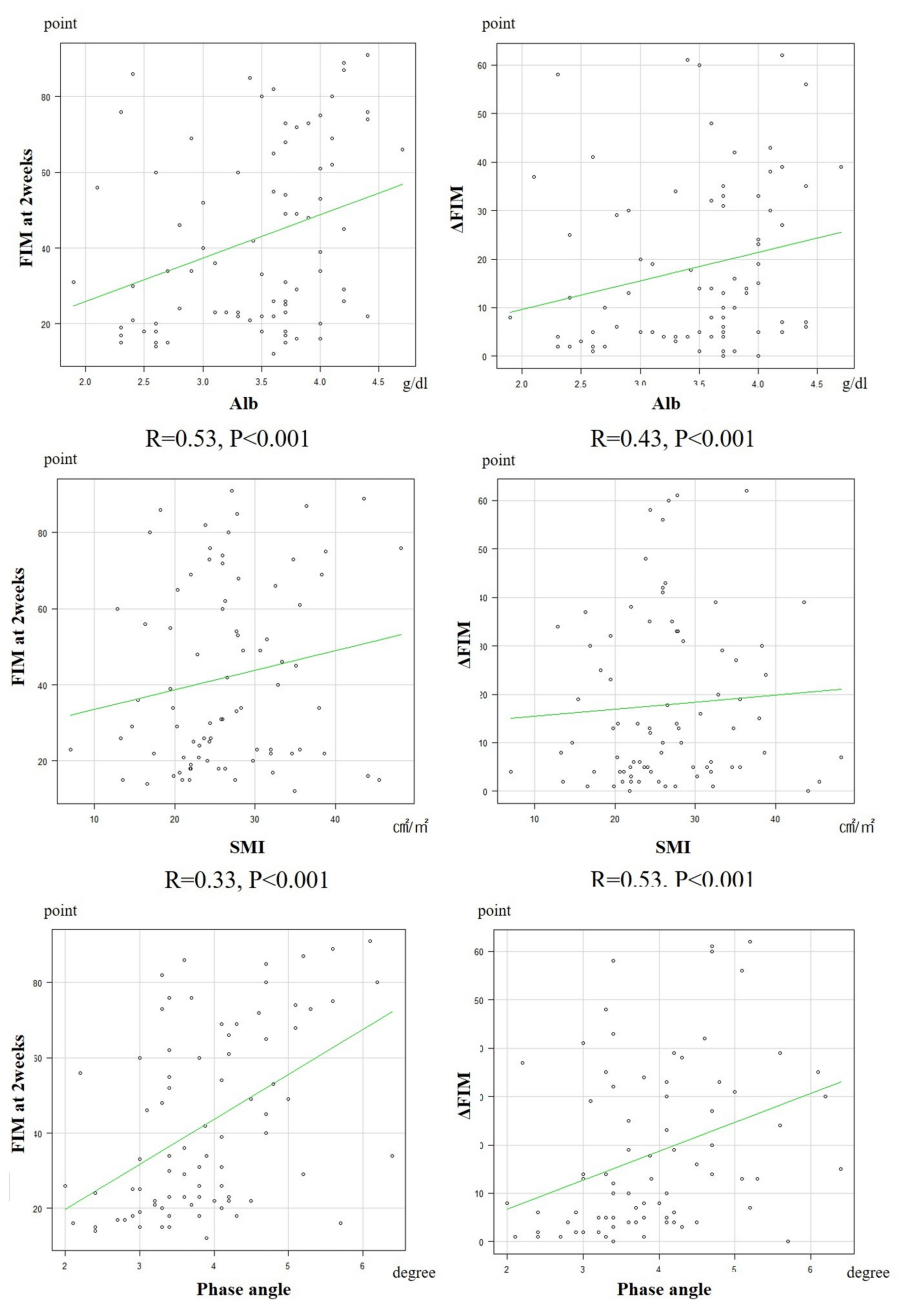
Correlations among serum albumin level, skeletal muscle index (SMI), the phase angle, and functional independence measure (FIM) scores (FIM at two weeks postoperatively and ΔFIM)

The mean FIM scores at admission were 24.3, the score at two weeks postoperatively was 42.0, and the mean ΔFIM was 17.8. Positive correlations were observed between the phase angle with both the FIM score at two weeks postoperatively (R=0.48, p<0.001) and ΔFIM (R=0.34, p<0.005). Additionally, positive correlations were observed between serum albumin level at admission and the FIM score at two weeks postoperatively (R=0.31, p<0.005) and ΔFIM (R=0.23, p<0.05). However, no correlations were observed between SMI with either the FIM score at two weeks postoperatively or ΔFIM (Figure 3).

From multiple regression analysis with the FIM score at two weeks postoperatively or ΔFIM as the dependent variable, and the phase angle, serum albumin level at admission, and SMI as three independent variables, only the phase angle showed significant correlations with the FIM score at two weeks postoperatively (p<0.001) and ΔFIM (p<0.05) (Table 2).

**Table 2:** Multiple regression analysis β = standardized regression coefficient; SE = standard error; t = t-value in regression analysis “Fracture type” indicates the anatomical classification of the hip fracture (femoral neck or trochanteric). “Complication” refers to the presence of postoperative medical or surgical adverse events during hospitalization.

Variables	FIM score at 2 weeks postoperatively				ΔFIM			
	β	SE	t	p	β	SE	t	p
Age	-0.511	0.360	-1.421	0.160	-0.693	0.271	-2.559	0.013*
BMI	-0.038	0.839	-0.047	0.964	-0.220	0.618	-0.355	0.723
Fracture type	4.901	4.899	1.000	0.320	6.355	3.636	1.748	0.085
Alb	2.116	4.354	0.486	0.628	1.419	3.189	0.445	0.658
Phase angle	7.532	3.596	2.095	0.040*	2.283	2.647	0.862	0.391
SMI	0.025	0.373	0.067	0.948	-0.171	0.275	-0.621	0.537
Complications	-9.165	5.722	-1.602	0.114	-2.874	4.194	-0.685	0.495
R2	0.239				0.194			

## Discussion

Malnutrition is a subject of intense research in the field of geriatrics [10-12] because it is very common among older individuals with hip fractures. Malnutrition negatively influences functional recovery after a fracture, increases healthcare expenditure, and is associated with high mortality rates. Koval et al. [13] showed that albumin level was predictive of hospitalization duration, in-hospital mortality, and delayed recovery of basic activities of daily living after hip fracture. These authors also reported that patients with preoperative albumin levels <3.5 g/dL were more likely to have an increased hospitalization duration or to die during hospitalization and were less likely to recover to their pre-fracture level of function, in terms of basic activities of daily living, as compared to patients with higher albumin levels. Similarly, Sim et al. reported that preoperative hypoalbuminemia was associated with poor postoperative functional outcomes and quality of life after hip fracture surgery [14].

In the past decade, BIA has been widely used to analyze body composition, including segmental skeletal muscle mass, with fast and non-invasive advantages [10,15]. Additionally, many BIA indicators were found to be valuable in helping clinicians improve disease diagnosis and prognosis assessment [16]. Nevertheless, the accuracy of BIA in estimating body composition can be compromised when tissue hydration is abnormal, such as in individuals with liver cirrhosis, renal dysfunction, heart failure, postoperative edema, or obesity [17]. This limitation arises because BIA assumes a uniform tissue composition, a constant cross-sectional area, and an even distribution of electrical current density [18].

Phase angle primarily reflects the permeability of cell membranes, suggesting that the trunk-specific phase angle may partially represent the quality of trunk skeletal muscle. Uemura et al. reported that older adults with reduced phase angle values had a higher risk of future falls compared with those showing relatively high values [19]. Moreover, Chen et al. showed that a low phase angle measured by BIA was significantly associated with an increased chance of femoral neck fracture in people aged >75 years [20]. Compared to survivors among critically ill patients, non-survivors had significantly lower phase angle, higher extracellular water/total body water (ECW/TBW), and higher % TBW/fat-free mass [21]. The phase angle in cancer patients serves as a predictor of poor health status and lower survival [22]. However, in patients with hip fractures, only one study has evaluated the association between the phase angle and functional outcomes after surgery. This report showed that a low phase angle of the non-fractured limb was independently associated with worse functional outcomes at rehabilitation discharge in patients undergoing surgery for hip fracture [23].

To our best knowledge, this is the second study to evaluate the association between the phase angle and functional outcomes after surgery for hip fracture. In this study, the phase angle at admission was positively correlated with serum albumin level at admission and SMI. This finding indicated that the phase angle could be an index of nutritional status and skeletal muscle mass. Similar to other reports, serum albumin level at admission was positively correlated with both the FIM score at two weeks postoperatively and ΔFIM. Moreover, positive correlations were observed between the phase angle with both the FIM score at two weeks postoperatively and ΔFIM. Multiple regression analysis showed that only the phase angle had a significant correlation with the FIM score at two weeks postoperatively and ΔFIM, indicating that the phase angle was a useful index that reflected functional recovery in patients with hip fractures.

The present findings suggest that phase angle may serve as a more comprehensive marker that integrates both nutritional status and muscle function, which are crucial for postoperative recovery. Unlike albumin or muscle mass alone, the phase angle appears to reflect the overall physiological resilience of elderly patients with hip fractures. Therefore, preoperative assessment of phase angle may help clinicians stratify patients at risk of delayed recovery and implement early nutritional or rehabilitative interventions.

The strengths of this study include the use of objective and quantitative parameters such as serum albumin, SMI, and BIA-derived phase angle, allowing for a multifaceted assessment of nutritional and muscular status.

This study has several limitations. First, it was retrospective and conducted at a single institution with a relatively small sample size, which may limit the generalizability of our findings. Second, bias may have been introduced by differences in surgical procedures, rehabilitation intensity, or comorbidities that were not fully controlled. Third, although BIA is a convenient and noninvasive method, its accuracy may be influenced by body fluid imbalance or edema. Future multicenter, prospective studies with larger samples are warranted to confirm our results. Despite these limitations, our findings highlight the potential utility of the phase angle as a practical marker in the preoperative assessment of elderly patients with hip fractures.

## Conclusions

In conclusion, the preoperative phase angle could accurately predict postoperative functional recovery and identify the impact of undernutrition and sarcopenia on clinical outcomes in patients with hip fractures. To improve clinical outcomes effectively, orthopedic surgeons should be aware of geriatric nutritional problems in patients with hip fractures. A comprehensive approach that combines nutritional management and rehabilitation may be pivotal to improving the clinical outcomes.
